# mTOR和PTEN在非小细胞肺癌组织中的表达及临床意义

**DOI:** 10.3779/j.issn.1009-3419.2010.07.11

**Published:** 2010-07-20

**Authors:** 亮 王, 绍发 许, 文涛 岳, 晓婷 赵, 丽娜 张, 玥 王

**Affiliations:** 1 101149 北京，北京市结核病胸部肿瘤研究所，北京市肿瘤分子生物学实验室肺癌分室，细胞生物学研究室 Department of Cellular and Molecular Biology, Beijing TB and Thoracic Tumor Research Institute, Beijing 101149, China; 2 101149 北京，北京市结核病胸部肿瘤研究所，北京市胸科医院胸外科 Department of Thoracic Surgery, Beijing Chest Hospital, Beijing 101149, China

**Keywords:** mTOR, PTEN, 逆转录聚合酶链反应, 肺肿瘤, mTOR protein, PTEN protein, Reverse transcriptase polymerase chain reaction, Lung neoplasms

## Abstract

**背景与目的:**

mTOR是调节细胞生长和增殖的重要信号转导分子，也是一种蛋白激酶。它通过活化下游的相关的效应蛋白发挥作用。在信号转导通路中*PTEN*基因可通过对该信号途径的负调控而抑制mTOR的活化。本研究通过分析mTOR信号转导途径中*mTOR*和*PTEN*基因在非小细胞肺癌（non-small cell lung cancer, NSCLC）组织中的表达和临床意义。

**方法:**

外科手术中获取65例NSCLC组织及30例癌旁组织，RT-PCR技术检测NSCLC组织及癌旁组织中*mTOR*和*PTEN*基因的表达水平。

**结果:**

mTOR在NSCLC组织中表达量（0.23±0.16）显著高于癌旁组（0.12±0.09）（*P* < 0.01），PTEN在NSCLC组织中表达量（0.19±0.28）显著低于癌旁组（0.53±0.28）（*P* < 0.01）。mTOR和PTEN与病人的性别、年龄、病理类型、淋巴结转移情况无关，与病人的肿瘤大小有关。

**结论:**

mTOR在NSCLC中被激活，PTEN在NSCLC组织表达缺失或减少，mTOR通路的激活和PTEN表达缺失在NSCLC发生发展中起到一定的作用。

## Abstract (en)

**Background and objective:**

It has been proved that mTOR was an important signal transduction molecular and protein kinase regulating cell growth and proliferation, and mTOR could activate the downstream protein effector. PTEN could negatively regulate mTOR signal pathway and inhibit its activity. The aim of this study is to detect the mRNA expression levels of *mTOR* and *PTEN* gene, which are the key genes of mTOR signaling pathway in human non-small cell lung cancer (NSCLC) tissue. The relationship between mTOR signaling pathway and NSCLC is also explored.

**Methods:**

Lung cancer tissue specimens were obtained from 65 patients. Adjacent-tumor non-small cell lung cancer tissues from the 30 patients were served as control. The RT-PCR technique was used to detect the *mTOR* and *PTEN* gene expression levels.

**Results:**

The average mRNA expression levels of mTOR gene were significantly higher (0.23±0.16) in lung cancer than in adjacent-tumor tissue (0.12±0.09)(*P* < 0.01). The average mRNA expression levels of *PTEN* gene were (0.19±0.28) in lung cancer, while the mRNA expression levels of *PTEN* gene were (0.53±0.28) in adjacent-tumor tissue (*P* < 0.01). The levels of *PTEN* gene expression in non-small cell lung cancer were significantly lower than that in adjacent-tumor lung tissue. There are not significant relationship between *mTOR* and *PTEN* gene expression levels and patients' age, gender, pathological type, differentiation, lymph node metastasis, except tumor size.

**Conclusion:**

The expression of mTOR is activated in NSCLC. The expression of PTEN is absent or decreased. The mTOR activated in NSCLC may be correlate with the absent or decreased of PTEN. The absent or decreased expression of PTEN and the actived mTOR may play important roles in carcinogenesis and metastasis of NSCLC.

肺癌是当今世界上严重威胁人类健康与生命的恶性肿瘤，我国卫生部2008年5月公布的数据表明，肺癌是我国恶性肿瘤中致死的首要因素。肺癌发生的分子机制尚未明了，癌基因的激活及抑癌基因的缺失和（或）突变以及DNA修复基因突变和（或）缺失是导致肺癌发生的主要原因。研究表明：哺乳动物雷帕霉素靶蛋白（mammal target of rapamycin, mTOR）是控制蛋白翻译和细胞周期进展的重要调控因子，处于生长调节的中心环节，它控制细胞内mRNA的翻译，参与膜蛋白转运、蛋白质降解、蛋白激酶C信号转导和核糖体合成等一系列生理病理过程。mTOR作为介导肿瘤细胞凋亡、增生、分化、代谢的重要信号传导通路，通路的失调可能对肺癌形成具有重要的作用^[1, 2]^。第10染色体丢失的磷酸酶基因（phosphatase and tensinhomologue deleted on chromosome 10, *PTEN*）是Li等^[3]^发现的同时具有脂质磷酸酶和蛋白磷酸酶双特异性磷酸酶活性的抑癌基因。这一基因定位于染色体10q23，有9个外显子，编码403个氨基酸组成的蛋白。在大多数肿瘤中包括肺癌，*PTEN*基因常有异常改变，提示*PTEN*基因异常在肿瘤的发生、发展过程中具有重要的作用。*PTEN*基因作为第一个被发现的具有双重特异性磷酸酶活性的肿瘤抑制基因，在细胞的生长发育、凋亡、迁移、信号转导等方面起着重要的调控作用。而突变的PTEN失去了对细胞生长的负调控，可导致肿瘤进行性生长^[4]^。PTEN功能丢失可激活mTOR信号传导通路，参与肿瘤的发生^[5]^。mTOR和PTEN二者与肿瘤的发生关系密切。因此，本实验以mTOR通路中的两个重要基因*mTOR*和*PTEN*为观察指标，通过检测NSCLC组织和癌旁组织中上述基因的表达变化，观察mTOR和PTEN的活化在非小细胞肺癌（non-small cell lung cancer, NSCLC）中的表达以及二者的关系。

## 1 材料与方法

### 1.1 材料

肺癌组织取自2007年4月-2008年10月北京胸科医院手术标本65例，鳞癌38例，腺癌27例，其中高分化5例，中分化43例，低分化例17例。临床分期，依据1997年国际抗癌联盟（UICC）TNM分期标准，其中Ⅰ期-Ⅱ期38例，Ⅲ期-Ⅳ期27例。全组男55例，女10例，中位年龄60岁，其中 < 60岁组31例，≥60岁组34例，年龄最大84岁，最小36岁。所有患者术前均未经任何化疗和抗肿瘤治疗，经临床及病理学检查确诊为NSCLC。同时取癌旁组织30例设为对照，进行配对研究。所有组织标本采集后迅速液氮保存备检。

### 1.2 *mTOR*、*PTEN*基因mRNA表达水平的测定

#### 1.2.1 试剂

Trizol、DEPC购自美国Gibco公司，dNTP、M-MLV逆转录酶、Taq酶、琼脂糖等为美国Promega公司产品，RNasina Ribonuclease Inhibitor购自华美生物工程公司，DNA Markers Ⅱ (100 bp、250 bp、500 bp、750 bp、1 000 bp、2 000 bp)购自北京Tiangen公司，引物由上海生工生物技术服务有限公司合成。

#### 1.2.2 方法

30 mg肺癌或正常组织，用Trizol一步法提取总RNA，经电泳显示28S、18S和5S清晰三条带，测定OD_260_/OD_280_比值1.8-2.0。采用逆转录聚合酶链反应（reverse transcriptase polymerase chain reaction, RT-PCR）检测*mTOR*、*PTEN*基因mRNA表达水平，两个基因的引物序列见表 1。RT反应体系20 μL，PCR反应体系25 μL。PCR扩增反应条件为：95 ℃预变性5 min，94 ℃变性30 s，X ℃退火30 s，72 ℃延伸40 s，循环35次，72 ℃终末延10 min。模板量及循环数经检测均在线性范围内。PCR产物琼脂糖凝胶电泳后，以Gel Pro Analyzer 4.0计算机图像扫描分析系统测定电泳条带密度值，以β-actin为内参照，计算*mTOR*、*PTEN*基因mRNA的相对含量，结果以mTOR、PTEN条带占β-actin条带中央点密度的百分率（%）表示。

**1:** 目标基因的引物设计 Primers for two genes

Gene	Up-primer/ Down-primer	Annealing temperature	Length
*β*-actin	CACTGTGTTGGCGTACAGGT	62.5 ℃	154 bp
	TCATCACCATTGGCAATGAG		
*mTOR*	CGCTGTCATCCCTTTATCG	56.5 ℃	193 bp
	ATGCTCAAACACCTCCACC		
*PTEN*	ACCAGGACCAGAGGAAACCT	55 ℃	232 bp
	GCTAGCCTCTGGATTTGACG		

### 1.3 统计学处理

采用SPSS 13.0与Excel软件进行数据整理和统计，结果以目标基因的相对表达量的Mean±SD表示，采用*t*检验分析目标基因在NSCLC组织和癌旁组织中，以及不同病例信息之间的表达量，*Kendall's taub*相关性分析目标基因相对表达量之间的相关性，*P* < 0.05为差异具有统计学意义。

## 2 结果

### 2.1 RNA纯度检测和浓度测定

将提取的肺癌组织及癌旁组织的总RNA进行凝胶电泳，结果显示5S、18S、28S三条清晰条带（图 1），表明所得RNA完整性较好；再经紫外分光光度仪确定所得RNA的*A*_260_/*A*_280_比值在1.8-2.0之间，说明RNA无蛋白污染，符合RT-PCR实验要求。最后，通过OD_260_值计算RNA浓度，进行逆转录反应。

**1:**
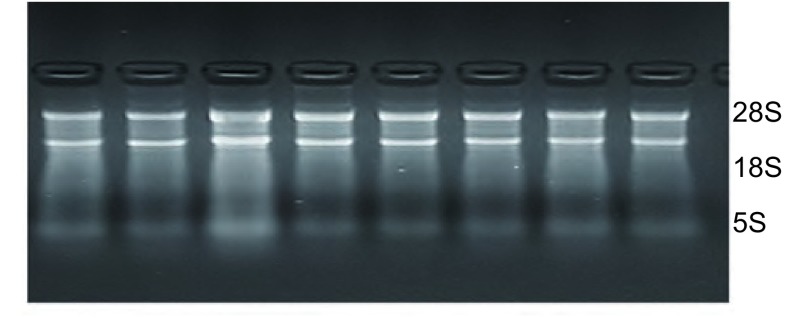
肺组织总RNA提取 Total RNA of lung tissues

### 2.2 癌组织及癌旁组织中*mTOR*、*PTEN*基因mRNA表达水平检测

研究中通过琼脂糖凝胶对PCR结果进行检测（图 2），肺癌与癌旁组织中β-actin表达水平一致，对电泳条带密度值进行统计分析：mTOR在NSCLC组织中表达量（0.23±0.16）显著高于癌旁组（0.12±0.09）（*P* < 0.01），PTEN在NSCLC组织中表达量（0.19±0.28）显著低于癌旁组（0.53±0.28）（*P* < 0.01）。

**2:**
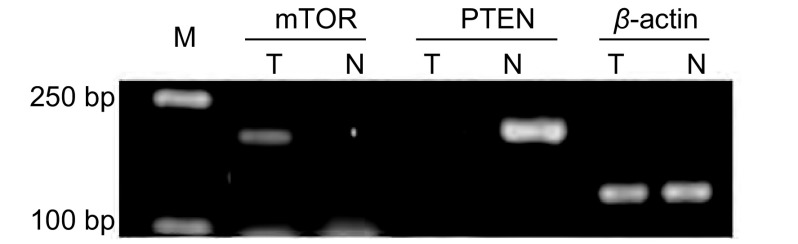
目标基因的扩增。T泳道为癌组织；N泳道为癌旁组织。 The amplification of target genes, mTOR、PTEN、*β*-actin are in order. T are from tumor tissues; N are from tumor adjacent tissues.

### 2.3 *mTOR*、*PTEN*基因的表达水平与NSCLC患者临床特征的关系

*mTOR*、*PTEN*基因的表达水平与NSCLC患者的年龄、性别、病理类型、分化程度、淋巴结转移、远处转移无明显关系，但与肿瘤大小有显著关系（表 2）。

**2:** 临床病理参数与两个基因表达之间的关系 The relationship of clinicopathological parameters and two geneexpressions

Characteristics	*n*	*P*	
		*mTOR*	*PTEN*
Age		0.471	0.273
< 60	31		
≥ 60	34		
Gender		0.810	0.974
Male	55		
Female	10		
Pathological type		0.235	0.124
Squamous	37		
Adenocarcinoma	28		
Differentiation		0.765	0.777
High	5		
Mild	43		
Low	17		
Tumor size		0.04	0.01
≤3 cm	15		
> 3 cm	50		
Lymph node metastasis		0.468	0.335
No	46		
Yes	19		
Distant metastases		0.496	0.958
No	61		
Yes	4		

### 2.4 mTOR与PTEN相关性分析

肺癌组织中*mTOR*、*PTEN*基因相对表达量的ROC曲线分析结果（图 3），AUC（area under the curve）值均 > 0.5，说明这些曲线具有诊断意义。*mTOR*、*PTEN*基因相对表达值的散点图cut-off值见图 4，虚线表示这两个基因为诊断标准。两个基因的cutoff值均为0.38，选定敏感性和特异性分别为64%/66%、89%/73%，当然此cut-off值也取决于敏感性特异性相对值的选取。使用SPSS 13.0软件进行*Kendall's taub*非参数相关性分析来研究两个基因的相对表达量之间的相关性，结果显示：mTOR与PTEN的相对表达量之间呈负相关。随着肿瘤的发展，PTEN的表达水平呈递减趋势。

**3:**
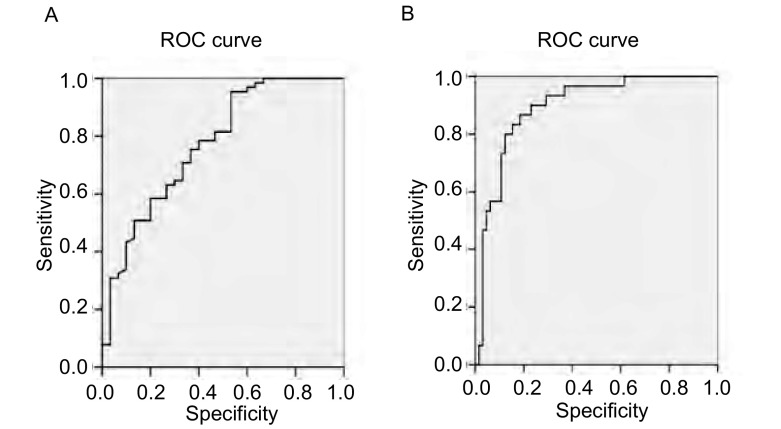
*mTOR*和*PTEN*基因表达量的曲线分析 The ROC curve analysis of the *mTOR*, *PTEN* gene expressions. A: The ROC curve analysis of the *mTOR* gene expression, AUC=0.771; B: The ROC curve analysis of the *PTEN* gene expression, AUC=0.894.

**4:**
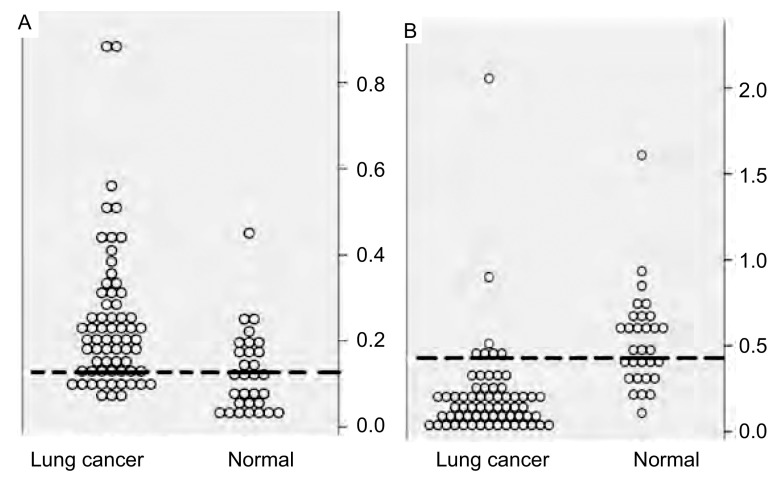
*mTOR*、*PTEN*基因相对表达水平的散点图 Scatter distribution of relative expressions of *mTOR*, *PTEN* gene. A: Scatter distribution of relative expression of *mTOR* gene; B: Scatter distribution of relative expression of *PTEN* gene.

## 3 讨论

NSCLC是当今最常见的恶性肿瘤之一，目前对NSCLC的治疗方法还是传统的手术、化疗和放疗，但是5年生存率仍仅为10%-15%，其中大部分病人经确诊时已是中晚期，失去了治疗机会。所以，早发现早治疗成为提高5年生存率的关键。近年来，随着分子生物学的进展，人们一直在寻找一种更好的肺癌标志物，mTOR信号通路在人类肿瘤的作用得到了广泛的关注。mTOR信号通路的活化在人类肿瘤的发生中极为普遍，该通路可以通过多种机制促进肿瘤的发生，包括基因突变、抑癌基因*PTEN*的表达减少、磷脂酰肌醇3激酶（phosphatidylinositol 3-kinase, PI3K）、蛋白激酶B（protein kinase B, AKT）的扩增、癌基因受体的活化等^[6, 7]^。mTOR在多种肿瘤组织中高表达，如卵巢癌、前列腺癌、多发性骨髓瘤、乳腺癌、胰腺癌、肺癌、子宫内膜癌等癌组织中均有mTOR的过度表达及活化^[8]^。*mTOR*基因在肺癌及侵袭前支气管病变中均表现为基因组的扩增，这提示mTOR通路与肺癌的发展有关^[9]^。*PTEN*基因主要通过调控信号传导和细胞周期来发挥抑癌作用，主要功能为抑制细胞生长，抑制细胞粘附、浸润与转移，促进细胞骨架形成，促进细胞凋亡和调节血管生成^[10]^。*PTEN*基因具有磷酸酶活性，其蛋白具有脱磷酸作用，磷脂酰肌醇3磷酸（phosphatidylinositol-3, PIP3）脱磷酸形成磷脂酰肌醇2磷酸（phosphatidylinositol-2, PIP2），使其丧失信使功能。

本实验结果显示，mTOR在NSCLC组织中表达量显著高于癌旁组，PTEN在NSCLC组织中表达量显著低于癌旁组织，与Lim等的报道一致^[11]^。在本实验中*mTOR*、*PTEN*基因的表达水平同NSCLC患者的肿瘤大小有显著关系，PTEN在NSCLC发展过程中的表达缺失呈现一种逐渐升高的进行性变化趋势，并逐渐丧失其抑癌功能。PTEN在Ⅲ期NSCLC中表达显著低于Ⅰ期NSCLC，提示*PTEN*基因表达缺失可能是NSCLC发展过程中的晚期事件之一。但近期有实验证实PTEN失活可加速小鼠肺癌模型中肿瘤的形成，从而提示肺癌形成早期即有PTEN失活。在本实验中*mTOR*、*PTEN*基因的表达水平同NSCLC患者的年龄、性别、病理类型、分化程度、淋巴结转移、远处转移无明显关系，与个别报道不符，究其原因可能与研究对象的临床病理特征组成不同、mTOR和PTEN阳性的判断方法不同有关。另外，在一些指标中*P*值接近0.05，考虑可能时由于本实验样本量较小，若加大样本量可能会有差异。PTEN的缺失或低表达失去了对mTOR的抑制作用，使得mTOR过度表达。两者共同促进NSCLC的发展，相关性分析表明：两者呈负相关。说明PTEN的失活与mTOR的高表达在NSCLC的生长增殖中起一定的作用，并且关系密切。PTEN通过抑制PIP3的活化，使肿瘤细胞的生长受到抑制，而mTOR的活化可促使该进程的发展。*PTEN*是一种抑癌基因，可以逆转mTOR所催化的反应，可介导的细胞增殖，是mTOR通路中的一个负向调节因子，PTEN的肿瘤抑制功能主要依赖其脂质磷酸酶的活性，它可通过特异性地促使PIP3的脱磷酸化，使之转化为PIP2，使PIP3无法激活下游的AKT，从而起不到抑制AKT/mTOR通路的活性的作用，mTOR通路活性增强，下游的核糖体蛋白S6（ribosomal protein S6, RPS6）、p70核糖体S6激酶（P70 ribosomal S6 kinase）、真核生物翻译起始因子4E结合蛋白（eukaryotic translation initiation factor 4E binding protein, 4EBP）等相关效应蛋白随之增强^[12]^。自*PTEN*基因被发现至今，其作为一种肿瘤抑制基因，PTEN在多种肿瘤中的高频率的杂合性丢失，被认为是后p53时代突变最多的肿瘤抑制基因，mTOR和PTEN在生长因子受体信号通路扮演着非常重要的角色，mTOR和PTEN的联合检测可作为诊断NSCLC的重要肿瘤标志物。
